# Acupotomy Contributes to Suppressing Subchondral Bone Resorption in KOA Rabbits by Regulating the OPG/RANKL Signaling Pathway

**DOI:** 10.1155/2021/8168657

**Published:** 2021-04-26

**Authors:** Tong Wang, Yan Guo, Xiao-Wei Shi, Yang Gao, Jia-Yi Zhang, Chun-Jiu Wang, Xue Yang, Qi Shu, Xi-Lin Chen, Xin-Yi Fu, Wen-Shan Xie, Yi Zhang, Bin Li, Chang-Qing Guo

**Affiliations:** ^1^School of Acupuncture-Moxibustion and Tuina, Beijing University of Chinese Medicine, Beijing 100029, China; ^2^Acupuncture and Moxibustion Department, Beijing Hospital of Traditional Chinese Medicine affiliated with Capital Medical University, Beijing 100010, China; ^3^Massage Department, The Third Affiliated Hospital of Beijing University of Chinese Medicine, Beijing 100029, China; ^4^Traditional Chinese Medicine Department, Beijing Nankou Hospital, Beijing 102200, China

## Abstract

Subchondral bone lesions, as the crucial inducement for accelerating cartilage degeneration, have been considered as the initiating factor and the potential therapeutic target of knee osteoarthritis (KOA). Acupotomy, the biomechanical therapy guided by traditional Chinese meridians theory, alleviates cartilage deterioration by correcting abnormal mechanics. Whether this mechanical effect of acupotomy inhibits KOA subchondral bone lesions is indistinct. This study aimed to investigate the effects of acupotomy on inhibiting subchondral bone resorption and to define the possible mechanism in immobilization-induced KOA rabbits. After KOA modeling, 8 groups of rabbits (4w/6w acupotomy, 4w/6w electroacupuncture, 4w/6w model, and 4w/6w control groups) received the indicated intervention for 3 weeks. Histological and bone histomorphometry analyses revealed that acupotomy prevented both cartilage surface erosion and subchondral bone loss. Further, acupotomy suppressed osteoclast activity and enhanced osteoblast activity in KOA subchondral bone, showing a significantly decreased expression of tartrate-resistant acid phosphatase (TRAP), matrix metalloproteinases-9 (MMP-9), and cathepsin K (Ctsk) and a significantly increased expression of osteocalcin (OCN); this regulation may be mediated by blocking the decrease in osteoprotegerin (OPG) and the increase in NF-*κB* receptor activated protein ligand (RANKL). These findings indicated that acupotomy inhibited osteoclast activity and promoted osteoblast activity to ameliorate hyperactive subchondral bone resorption and cartilage degeneration in immobilization-induced KOA rabbits, which may be mediated by the OPG/RANKL signaling pathway. Taken together, our results indicate that acupotomy may have therapeutic potential in KOA by restoring the balance between bone formation and bone resorption to attenuate subchondral bone lesions.

## 1. Introduction

Although cartilage degeneration is the most typical pathological feature of knee osteoarthritis (KOA) [1]; the critical role of subchondral bone lesions in KOA has gradually become an area of focus in recent years [2, 3]. Increasing evidence implies that subchondral bone lesions may be the initiating factor in KOA [4, 5]. Subchondral bone lesions were accompanied by a reduced ability of cushioning mechanical stress, which induced and accelerated the overlying cartilage degeneration under abnormal mechanical stress [6, 7]. Clinical studies have confirmed that inhibiting subchondral bone lesions can effectively relieve cartilage erosion [8, 9], which implied that subchondral bone was a potential therapeutic target for protecting cartilage and treating KOA.

During the course of KOA, persistent abnormal load within the joint leads to subchondral bone microfractures, which initiate bone remodeling [10]. Bone remodeling is the result of disrupting the balance between bone formation and bone resorption. In the early-stage KOA, due to the abnormal activation of osteoclasts [11] in the subchondral bone, bone remodeling is mainly characterized by hyperactive bone resorption [12]. Osteoprotegerin (OPG)/NF-*κB* receptor activated protein ligand (RANKL) is the critical pathway to regulate bone remodeling [13, 14], which is mainly realized by regulating osteoclast activity. RANKL binds to RANK to accelerate osteoclast precursor differentiation and promote osteoclast maturation [15]. OPG can inhibit osteoclast activity by blocking the binding of RANK and RANKL [16]. Studies confirmed that bone resorption can be effectively alleviated by regulating OPG/RANKL pathway [17, 18].

Inhibition of subchondral bone resorption as a therapeutic target for KOA has been carried out extensively. Bone loss inhibitors, such as bisphosphonates, are potentially effective modalities for KOA treatment by inhibiting subchondral bone resorption [19]. However, the latest meta-analysis showed that the clinical efficacy of bisphosphonates remains to be verified [20]. Thus, nonpharmacological therapies to inhibit subchondral bone resorption deserve extensive investigation.

Acupotomy is a biomechanical therapy under the guidance of traditional Chinese meridians theory. Acupotome, also referred to as miniscalpel-needle acupuncture, is a fairly recent development in the practice of acupuncture. Acupotome combines the characteristics of acupuncture needle insertion and surgical incision and has a definite clinical effect on KOA. The biomechanical effect of acupotomy is the unique superiority that distinguishes it from other KOA therapies. Preliminary studies [21–24] have confirmed that acupotomy can prevent cartilage degeneration and delay KOA pathological procession by correcting the abnormal mechanical stress in KOA joints. Is the chondroprotective effect of acupotomy related to the inhibition of KOA subchondral bone resorption? Is it mediated by the OPG/RANKL signaling pathway to regulate osteoclast activity in subchondral bone?

Based on this hypothesis, we established the KOA model rabbits with Videman's extended immobilization method to explore whether acupotomy intervention could alleviate KOA subchondral bone lesions by inhibiting bone resorption, to observe the changes in activity biomarkers of osteoclasts and to evaluate the effect of the OPG/RANKL signaling pathway in subchondral bone.

## 2. Methods

### 2.1. Ethics Statement

Animal experiments were strictly handled in conformity to the recommendations in the Guidance Suggestions for the Care and Use of Laboratory Animals of Beijing University of Traditional Chinese Medicine (Beijing, China). The protocol was approved by the Committee on the Ethics of Animal Experiments of Beijing University of Traditional Chinese Medicine. All operations are carried out under pentobarbital anesthesia to ensure that pain was minimized.

### 2.2. Reagents and Materials

Disposable acupuncture needles (specification: 0.25 × 25 mm) were purchased from Beijing Research Taihe Pharmaceutical Co., Ltd. (Beijing, China). The HZ series disposable acupotome (specification: 0.4 × 40 mm) was purchased from Beijing Outstanding Huayou Medical Instrument Co., Ltd. (Beijing, China). Resin bandages (5#: 150 mm × 1800 mm) were purchased from Hengshui Kangjie Medical Instrument Co., Ltd. (Hebei, China). Orthopedic casting tapes (160 series tape: 5.0 cm × 360 cm) were purchased from Suzhou Connect Medical Technology Co., Ltd. (Jiangsu, China). The HANS acupoint nerve stimulator (LH202H) was purchased from Beijing Huawei Industry Development Co., Ltd. (Beijing, China).

Anti-OCN, anti-MMP-9, and anti-GAPDH antibodies were purchased from Abcam Biotechnology Co. Ltd. (USA). Anti-OPG and anti-RANKL antibodies were purchased from Bioss Biotechnology Co. Ltd. (USA). Horseradish peroxidase- (HRP-) conjugated secondary antibodies were provided by Beijing Zhongshan Golden Bridge Biotechnology Co. Ltd. (Beijing, China). Bicinchoninic acid (BCA) protein assay kit and Chemiluminescence ECL reagent were provided by Beyotime Biotechnology Co. Ltd. (Shanghai, China). The mouse polymer method detection kit was purchased from Beijing Zhongshan Golden Bridge Biotechnology Co. Ltd. (Beijing, China). TRIzol Reagent and Reverse Transcription kit were provided by Thermo Fisher Biotechnology Co. Ltd. (USA). PCR SYBR Green Master Mix was provided by Promega Biotechnology Co. Ltd. (USA). The commercial TRAP kit was provided by Sigma Biotechnology Co. Ltd. (Missouri, USA).

### 2.3. Animals and Study Design

Fifty-six male New Zealand white rabbits (6 months old) weighing approximately 2.5 kg were provided by the Laboratory Animal Center of Beijing Keyu Technology Co., Ltd. (Beijing, China). Rabbits were acclimatized for one week before the experiment began and were given unlimited food and water. Animals were raised in separate cages, with the temperature and humidity maintained at (22 ± 2)°C and 50%–60%, respectively. The rabbits were randomly divided into 8 groups as follows: KOA induced by immobilization for 4 or 6 weeks with vehicle treatment (4w model group, *n* = 7; 6w model group, *n* = 7), blank control group compared with the 4w or 6w Model group with vehicle treatment (4w control group, *n* = 7; 6w control group, *n* = 7), KOA induced by immobilization for 4 weeks or 6 weeks with acupotomy treatment (4w acupotomy group, *n* = 7; 6w acupotomy group, *n* = 7), and KOA induced by immobilization for 4 weeks or 6 weeks with electroacupuncture treatment (4w electroacupuncture group, *n* = 7; 6w electroacupuncture group, *n* = 7).

### 2.4. Induction of KOA Rabbits

The modified Videman method was performed to induce the KOA model. Before the operation, each rabbit was fasted and deprived of water for 10 to 16 hours; then, the rabbits were anesthetized by intravenous injection of 3% sodium pentobarbital (30 mg/kg) into the ear marginal vein. After anesthesia was completed, each rabbit was posited supinely on the operating table with the left hind leg exposed. The left hind leg was fixed with the resin bandage from the groin to the toe to maintain the extended position of the knee joint, and additional double layers of orthopedic casting tapes were wrapped to reinforce the fixation; the antibiting bandage was then applied at the outermost layer. During the immobilization period, extremity swelling and mold shedding were observed at all times, adjusting and refixing as necessary. The KOA model was established after effective immobilization for 4 weeks in the 4w model group, the 4w acupotomy group, and the 4w electroacupuncture group, and for 6 weeks in the 6w model group, the 6w acupotomy group, and the 6w electroacupuncture group. Rabbits in the 4w/6w control group did not undergo any operation.

### 2.5. Acupotomy and Electroacupuncture Interventions

After removing the bandages in the 4w/6w acupotomy groups, acupotomy intervention was performed once a week for 3 weeks. We selected four points as the fixed intervention points, located at the tendons of vastus medialis, vastus lateralis, rectus femoris, and biceps femoris. In addition, 1-2 knot nodes of muscle fibers in the extensor and flexor groups around the knee joint were selected according to individual conditions. After routine disinfection, the acupotome was pierced to release these points. The specific operations on each point were as follows. (1) The acupotome was inserted into the tendons by vertical insertion into the skin, and the blade of the acupotome was parallel to the longitudinal axis of the tendons. The local adhesion to the direction of tendons and bone connection was released by longitudinal dredging and transverse stripping, with 1-2 strikes per point; then, pressing the points for several minutes after the acupotome was withdrawn. (2) The acupotome was inserted into the knot nodes of muscle fibers by searching and locating the knot nodes of muscle fibers around the knee joint by palpation. The acupotome was inserted into the skin vertically, and the blade of the acupotome was parallel to the longitudinal axis of the muscle fibers. The local adhesion was released by longitudinal dredging and transverse stripping, with 1-2 strikes per point; then, pressing the points for several minutes after the acupotome was withdrawn.

After removing the bandages in the 4w/6w electroacupuncture groups, electroacupuncture intervention was performed every other day for 3 weeks. We selected four acupoints as the needle insertion points, namely, Xuehai (SP10), Liangqiu (ST34), Neixiyan (EX-LE4), and Waixiyan (EX-LE5). After routine disinfection, the needles were inserted into the four acupoints. Electroacupuncture intervention was performed with Han's acupoint nerve stimulator, which connected SP10 and ST34, EX-LE4 and EX-LE5, respectively. Waveforms with dense and dense waves: frequency 2/100 Hz, intensity 3 mA, 15 min each. In the 4w/6w control groups and 4w/6w model groups, only grabbing and fixing were administered. Animals were humanely sacrificed after the indicated intervention.

### 2.6. Cartilage Histology and Mankin Score

The cartilage-subchondral bone complex samples of the central weight-bearing area of the femur were fixed in 4% paraformaldehyde (Boster, Wuhan, China) for 24 hours at 4°C. All the samples were decalcified in neutral 10% EDTA (Boster, Wuhan, China) for 4 weeks. All samples were embedded in paraffin after dehydration with gradient alcohol and submersion in xylene and paraffin. The paraffin-embedded samples were sectioned from the sagittal position into 5 *μ*m thick sections. For histological analysis of cartilage, the sections were stained with safranin O and fast green (Solarbio, Beijing, China). The sections were evaluated for pathology of cartilage degeneration with a modified Mankin scoring system [25] by a double-blinded, independent pathologists.

### 2.7. Subchondral Bone Morphometric Parameter Calculation

To analyze the morphometric parameters of subchondral bone, the sections were stained with hematoxylin and eosin (HE) (Solarbio, Beijing, China) using standard protocols. Five fields were taken from each section and photographed under an optical microscope. Bone tissue static parameters including tissue area (T.Ar), trabecular area (Tb.Ar), and trabeculae perimeter (Tb.Pm) were performed using Image pro plus 6.0 (IPP) software. According to the T.Ar, Tb.Ar, and Tb.Pm data, the following parameters were quantified to represent the bone structure: percent of trabecular area (BV/TV), trabecular number (Tb.N), and trabecular separation (Tb.Sp) using bone histomorphometry [26].

### 2.8. Tartrate-Resistant Acid Phosphatase (TRAP) Staining

The osteoclast biomarker TRAP was applied to visualize the activity of osteoclasts. The sections were stained with a commercial TRAP kit according to the standard protocols. In general, after dewaxing with xylene and reconstituting with gradient ethanol, the sections were fixed with TRAP fixative solution at 4°C for 90 seconds and then incubated with an AS-BI Buffer, GBC staining solution, and TRAP Buffer mixture at 37°C for 60 min. Nuclei were counterstained for 2 min in hematoxylin solution. Three continuous sections from one sample were stained, and five fields were randomly selected from each section. TRAP-positive stained cells were quantified in subchondral bone regions.

### 2.9. Western Blot Analysis of OPG and RANKL

All soft tissues were stripped from the tibia. In the central weight-bearing area of the tibia, the cylindrical cartilage-subchondral bone complex tissues (∅ = 8 mm, Height = 10 mm) were intercepted with the circular drill. The cartilage was quickly separated from the subchondral bone at low temperature with a bone rongeur, and then the samples were quickly placed in liquid nitrogen. Total proteins were extracted from the subchondral bone using ultrasonication in RIPA lysis buffer containing 1% protease inhibitor cocktails (Beyotime Biotechnology, Shanghai, China), and then the protein concentrations were calculated with a bicinchoninic acid (BCA) protein assay kit (Beyotime Biotechnology, Shanghai, China). Equal quantities (50 *μ*g) of proteins were separated by electrophoresis using 12% sodium dodecyl sulfate-polyacrylamide electrophoresis (SDS-PAGE) (Solarbio, Beijing, China) gels and then transferred to 0.45 *μ*m PVDF membranes (Millipore, USA). Subsequently, the membranes were blocked with 5% nonfat milk for 2 hours at room temperature (RT) and then incubated with primary antibodies against OPG (1 : 500), RANKL (1 : 500), and GAPDH (1 : 1000) on a shaker at 4°C overnight. After washing in TBST, the membranes were incubated with horseradish peroxidase- (HRP-) conjugated secondary antibodies for 1 hour at RT. Finally, the bands were emerged with chemiluminescence ECL reagent. The grayscale value of the protein bands was analyzed using Image J software (Rockville, USA).

### 2.10. Real-Time PCR Analysis of OPG, RANKL, and Ctsk

The method of obtaining subchondral bone samples was the same as described above. Total RNA was extracted from the subchondral bone using TRIzol Reagent. RNA was reverse-transcribed into cDNA using the reverse transcription kit. For real-time PCR, cDNA amplification was handled using qPCR SYBR Green Master Mix. Based on the published sequences, specific PCR primers for OPG, RANKL, and Cathepsin K (Ctsk) were constructed. The relative expression of the target genes was calculated by the 2^−ΔΔ*Ct*^ method and normalized to the mRNA expression of GAPDH. Primer pairs were 5′-ATCATTGAATGGACAACCCAGG-3′ and 5′-TGCGTGGCTTCTCTGTTTCC-3′ for OPG, 5′-GGTTCCCATAAAGTGAGTCTGT-3′ and 5′-TTAAAAGCCCCAAAGTATG-3′ for RANKL, and 5′-CTTCCAATACGTGCAGCAGA-3′ and 5′-TCTTCAGGGCTTTCT-CGTTC-3′ for Ctsk, and 5′-CCACTTTGTGAAGCTCATTTCCT-3′ and 5′-TCGTCCTCCTCTGGTGCTCT-3′ for GAPDH, respectively.

### 2.11. Immunohistochemistry Analysis of OPG, RANKL, OCN, and MMP-9

Paraffin-embedded samples were prepared to detect the distribution of OPG, RANKL, osteocalcin (OCN) and matrix metalloproteinases-9 (MMP-9) in subchondral bone. Immunohistochemical staining was carried out using standard protocols. Briefly, the sections sustained the heat-induced antigen retrieval in citrate buffer (Solarbio, Beijing, China) for 15 min, incubated with anti-OPG, anti-RANKL, anti-OCN, or anti-MMP-9 primary antibodies overnight, incubated with HRP-conjugated secondary antibody for 20 min, and developed using the chromogenic reaction (brown) with DAB (Solarbio, Beijing, China) in the dark for 5–8 min. Five fields were randomly selected for each section. The sections stained with OPG and RANKL were counted to determine the number of positively stained cells, and the average optical densities of OCN and MMP-9 were calculated.

### 2.12. Statistical Analysis

Statistical analysis was performed with SPSS software (version 20.0, SPSS, Inc., Chicago, IL, USA) and was presented as the mean ± SD. Multiple comparisons of differences between groups were achieved by the least significant difference (LSD) test, and those among multiple groups were assessed by performing one-way ANOVA. Differences were regarded as statistically significant at *P* < 0.05 and *P* < 0.01.

## 3. Results

### 3.1. Acupotomy Intervention Alleviated KOA Cartilage Damage

**Figure 1:**
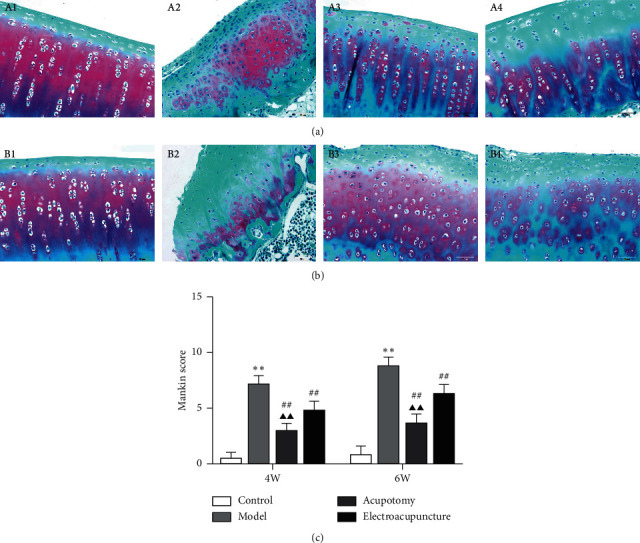
Acupotomy intervention alleviated KOA cartilage damage. (a) (b) Staining of cartilage with Safranin O-Fast green (magnification × 400). A1: 4w control group, A2: 4w model group, A3: 4w acupotomy group, A4: 4w electroacupuncture group; B1: 6w control group, B2: 6w model group, B3: 6w acupotomy group, B4: 6w electroacupuncture group. (c) Analysis of the Mankin score. Values are means ± SEMs. *n* = 6 per group. Compared with the corresponding control group: ^*∗*^*P* < 0.05 and ^*∗∗*^*P* < 0.01; compared with the corresponding model group: ^#^*P* < 0.05 and ^##^*P* < 0.01; compared with the corresponding electroacupuncture group: ^▲^*P* < 0.05 and ^▲▲^*P* < 0.01.

Safranin O-Fast Green staining detected no pathological erosion in the superficial cartilage in the 4w/6w control group (Figures 1(A1) and 1(B1)). In the 4w model group, the content of proteoglycan decreased in the cartilage, which was manifested by the partial deletion of Safranin O staining. Obvious cracks, fibrosis, and a disordered arrangement of chondrocytes were detected in the superficial cartilage (Figure 1(A2)). In the 6w model group with extensive exfoliation, prominent reduction of chondrocytes, continuous extension of fractures, and marked atrophy and necrosis of chondrocytes, large-scale deletion of Safranin O staining was detected in the superficial cartilage (Figure 1(B2)). The reduced deletion of Safranin O staining, relief of the disordered chondrocyte arrangement, and few cracks and fibrosis in the superficial cartilage were observed in the 4w/6w acupotomy compared with the corresponding 4w/6w model group (Figures 1(A3) and 1(B3)). No significant changes in the deletion of Safranin O staining, relief of disordered chondrocyte arrangement, reduction of chondrocyte loss, atrophic chondrocytes and cracks in the superficial cartilage were observed in the 4w/6w electroacupuncture compared with the corresponding 4w/6w model group (Figures 1(A4) and 1(B4)). However, deletion of Safranin O staining was significantly reduced in the 4w/6w acupotomy groups compared with the corresponding 4w/6w electroacupuncture groups. Moreover, the Mankin score results revealed that it was significantly increased in the 4w/6w model group (*P* < 0.01) compared with the corresponding 4w/6w control group, while it was significantly decreased (*P* < 0.01) in the 4w/6w acupotomy and electroacupuncture compared with the corresponding 4w/6w model groups (Figure 1(c)). Furthermore, it was significantly decreased (*P* < 0.01) in the 4w/6w acupotomy compared with the 4w/6w electroacupuncture groups (Figure 1(c)). These results suggested that both acupotomy and electroacupuncture interventions alleviated the cartilage damage in the KOA rabbits. The earlier the intervention, the better the effect. Moreover, the protective effect of acupotomy on KOA cartilage was better than that of electroacupuncture.

### 3.2. Acupotomy Intervention Inhibited Subchondral Bone Loss in KOA Rabbits

**Figure 2:**
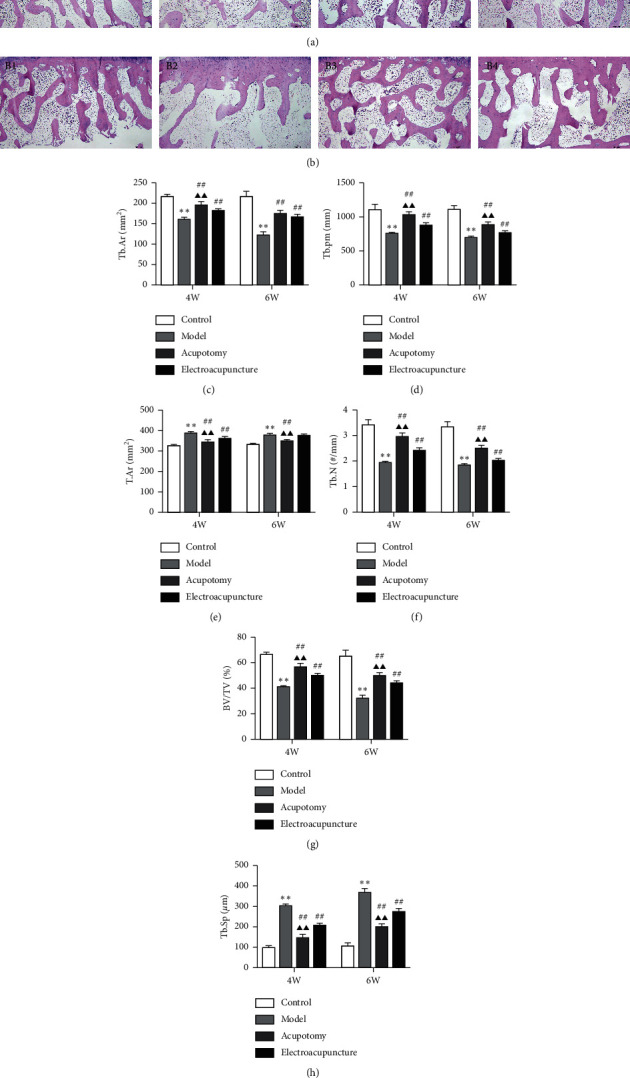
Acupotomy intervention inhibited subchondral bone loss in KOA rabbits. (a) (b) HE staining of subchondral bone (magnification × 40). A1: 4w control group, A2: 4w model group, A3: 4w acupotomy group, A4: 4w electroacupuncture group; B1: 6w control group, B2: 6w model group, B3: 6w acupotomy group, B4: 6w electroacupuncture group. (c) Analysis of Tb.Ar in subchondral bone. (d) Analysis of Tb.pm in subchondral bone. (e) Analysis of T.Ar in subchondral bone. (f) Analysis of Tb.N in subchondral bone. (g) Analysis of BV/TV in subchondral bone. (h) Analysis of Tb.Sp in subchondral bone. Values are means ± SEMs. *n* = 6 per group. Compared with the corresponding control group: ^*∗*^*P* < 0.05 and ^*∗∗*^*P* < 0.01; compared with the corresponding model group: ^#^*P* < 0.05 and ^##^*P* < 0.01; compared with the corresponding electroacupuncture group: ^▲^*P* < 0.05 and ^▲▲^*P* < 0.01.

The subchondral bone morphometric parameters calculated by HE stained sections are presented in Figures 2(a) and 2(b). Tb.Ar, Tb.Pm, Tb.N, and BV/TV were markedly decreased, and Tb.Sp was markedly increased in the 4w/6w model group (*P* < 0.01) compared with the corresponding 4w/6w control group, while Tb.Ar, Tb.Pm, Tb.N, and BV/TV were significantly increased (*P* < 0.01) and Tb.Sp significantly decreased in the 4w/6w acupotomy and electroacupuncture compared with the corresponding 4w/6w model groups. Moreover, except for Tb.Ar in the 6w acupotomy group, Tb.Ar, Tb.Pm, Tb.N, and BV/TV were markedly increased and Tb.Sp markedly decreased in the 4w/6w acupotomy group (*P* < 0.01) compared with the corresponding 4w/6w electroacupuncture group (Figures 2(c), 2(d), and 2(f)–2(h)). These data indicated that in KOA rabbits induced by immobilization for 4 weeks, lesions began to appear in the microstructure of subchondral bone, mainly manifested as trabecular destruction and bone mass reduction, which were gradually aggravated until model establishment for 6 weeks. After acupotomy or electroacupuncture intervention, the relevant parameters of subchondral bone structure were significantly altered, indicating that both interventions could effectively inhibit the subchondral bone resorption of KOA rabbits. Apparently, acupotomy can better inhibit the hyperactive bone resorption in KOA subchondral bone.

### 3.3. Acupotomy Intervention Enhanced Osteoblast Activity in KOA Subchondral Bone

**Figure 3:**
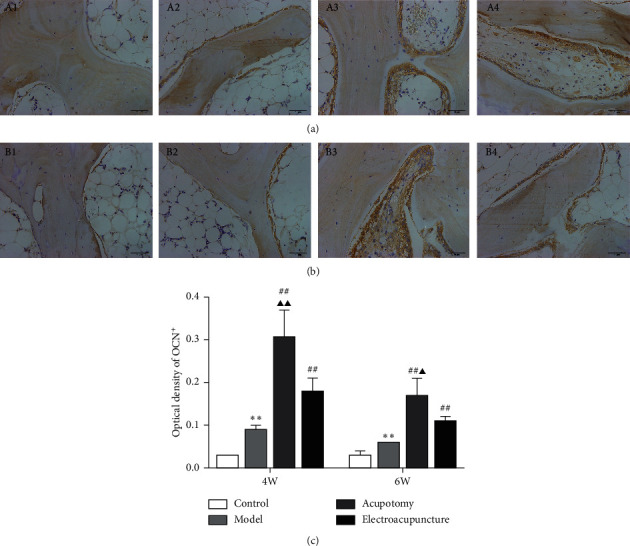
Acupotomy intervention enhanced the expression of OCN in KOA subchondral bone. (a, b) Immunohistochemical staining of OCN (magnification × 400). A1: 4w control group, A2: 4w model group, A3: 4w acupotomy group, A4: 4w electroacupuncture group; B1: 6w control group, B2: 6w model group, B3: 6w acupotomy group, B4: 6w electroacupuncture group. (c) Optical density values of OCN. Values are means ± SEMs. *n* = 6 per group. Compared with the corresponding control group: ^*∗*^*P* < 0.05 and ^*∗∗*^*P* < 0.01; compared with the corresponding model group: ^#^*P* < 0.05 and ^##^*P* < 0.01; compared with the corresponding electroacupuncture group: ^▲^*P* < 0.05 and ^▲▲^*P* < 0.01.

Immunohistochemical analysis detected low expression of OCN in subchondral bone in the 4w/6w control group (Figures 3(A1) and 3(B1)). Both 4w and 6w model groups showed higher expression of OCN (*P* < 0.01) compared with the 4w and 6w control group (Figures 3(A2) and 3(B2)). As expected, the expression of OCN in the 4w/6w acupotomy and electroacupuncture groups was markedly higher (*P* < 0.01) than in the corresponding 4w/6w model group, while it was significantly increased (*P* < 0.01, *P* < 0.05) in the 4w/6w acupotomy group compared with the 4w/6w electroacupuncture group (Figures 3(A3)-3(A4) and 3(B3)-3(B4)). These results indicated that OCN was compensatorily activated in the KOA damaged subchondral bone. The upregulation of OCN after the acupuncture or electroacupuncture intervention indicated that both delayed bone resorption by enhancing osteoblast activity of KOA subchondral bone. Acupotomy intervention could clearly better enhance osteoblast activity by upregulating OCN expression in KOA subchondral bone.

### 3.4. Acupotomy Intervention Suppressed the Activity of Osteoclasts in KOA Subchondral Bone

**Figure 4:**
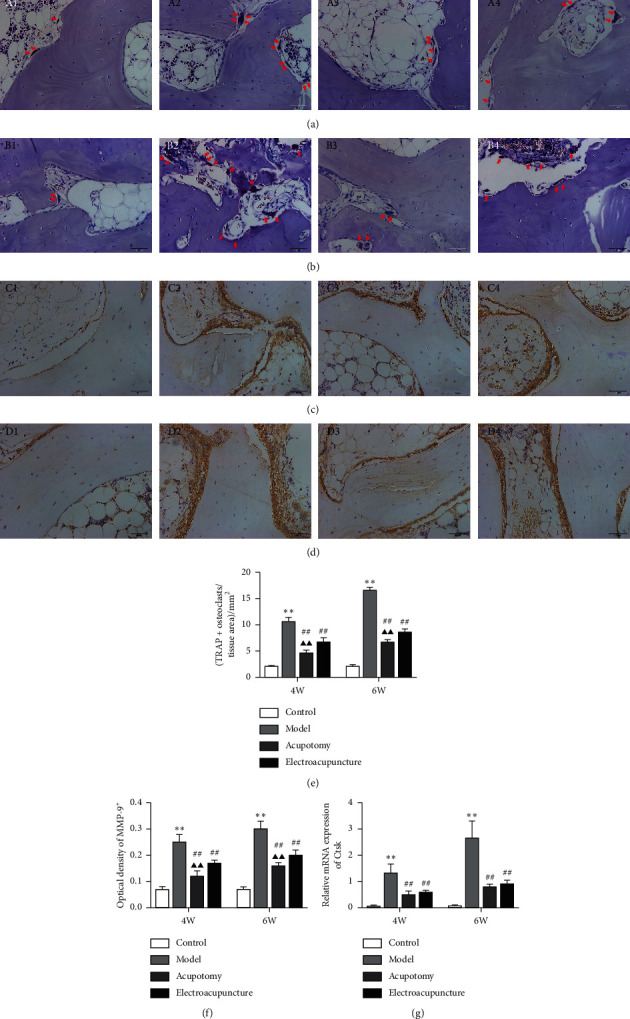
Acupotomy intervention suppressed the expression of TRAP, MMP-9, and Ctsk in KOA subchondral bone. (a, b) TRAP staining (magnification × 400). A1: 4w control group, A2: 4w model group, A3: 4w acupotomy group, A4: 4w electroacupuncture group; B1: 6w control group, B2: 6w model group, B3: 6w acupotomy group, B4: 6w electroacupuncture group. (c, d) Immunohistochemical staining of MMP-9 (magnification × 400). C1: 4w control group, C2: 4w model group, C3: 4w acupotomy group, C4: 4w electroacupuncture group; D1: 6w control group, D2: 6w model group, D3: 6w acupotomy group, D4: 6w electroacupuncture group. (e) The analysis of TRAP-positive cells in subchondral bone. (f) Optical density values of MMP-9. (g) Real-time PCR analysis of Ctsk. Values are means ± SEMs. *n* = 6 per group. Compared with the corresponding control group: ^*∗*^*P* < 0.05 and ^*∗∗*^*P* < 0.01; compared with the corresponding model group: ^#^*P* < 0.05 and ^##^*P* < 0.01; compared with the corresponding electroacupuncture group: ^▲^*P* < 0.05 and ^▲▲^*P* < 0.01.

The MMP-9 immunohistochemical staining results were consistent with the TRAP staining results. TRAP-positive and MMP-9-positive cells were occasionally observed in the subchondral bone in the 4w/6w control group (Figures 4(A1)–4(D1)). The number of TRAP-positive cells and MMP-9-positive cells in subchondral bone was significantly increased in the 4w/6w model group (*P* < 0.01) compared with the corresponding 4w/6w control group. It was apparent from the results that the number of TRAP-positive and MMP-9-positive cells increased in a more pronounced manner in the 6w model group, indicating the occurrence of more robust subchondral bone resorption (Figures 4(A2)–4(D2)). Both the 4w/6w acupotomy and electroacupuncture groups showed significantly lower (*P* < 0.01) expression of TRAP and MMP-9 compared with the corresponding 4w/6w model groups. Moreover, the expression of TRAP and MMP-9 in the 4w/6w acupotomy group was markedly lower (*P* < 0.01) than in the corresponding 4w/6w electroacupuncture group (Figures 4(A3)–4(D3) and 4(A4)–4(D4)). The expression of Ctsk mRNA further confirmed the results. However, there was no difference (*P* > 0.05) in the expression of Ctsk mRNA between the 4w/6w acupotomy and electroacupuncture groups (Figure 4(g)). These results indicated that TRAP, MMP-9, and Ctsk were overexpressed in the subchondral bone of immobilization-induced KOA rabbits, which signified that the osteoclasts were highly active and that bone remodeling had occurred mainly with bone resorption. By downregulating the expression of TRAP, MMP-9, and Ctsk, acupotomy or electroacupuncture intervention inhibited the activity of osteoclasts to suppress subchondral bone resorption.

### 3.5. Acupotomy Intervention Regulated the OPG/RANKL Pathway in KOA Subchondral Bone

**Figure 5:**
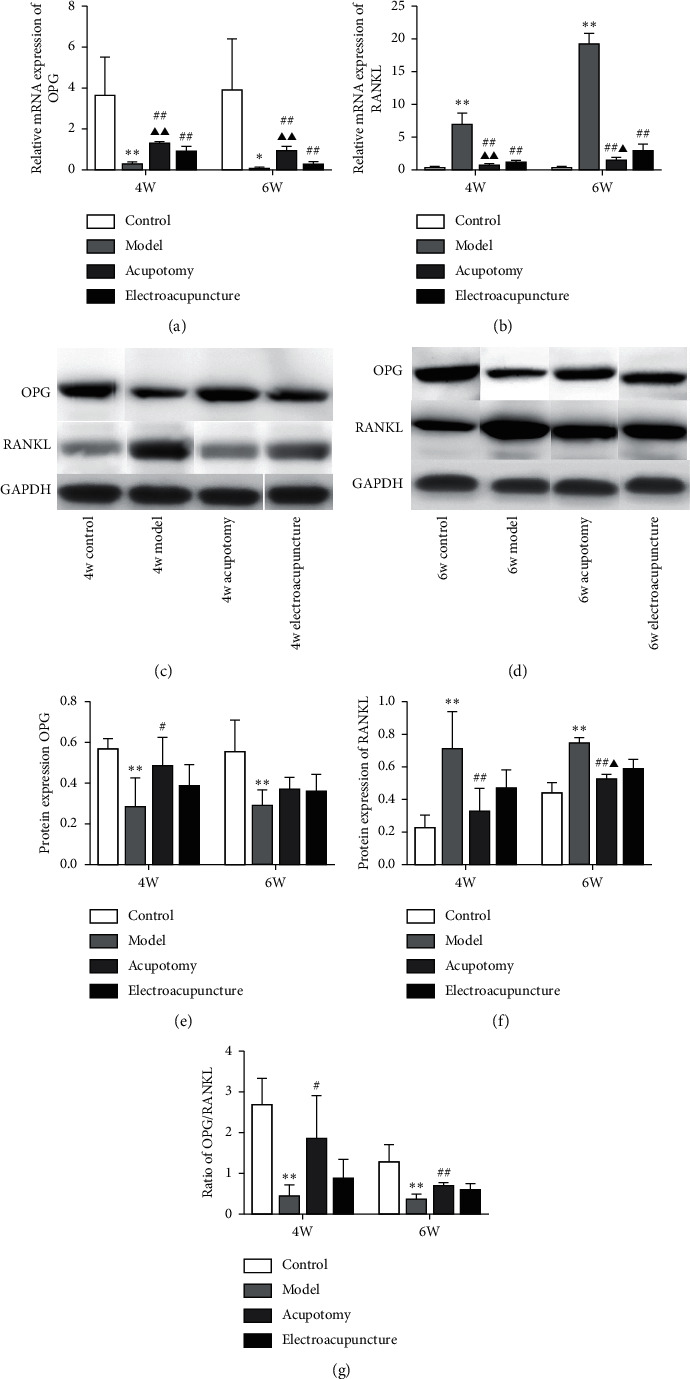
Acupotomy intervention upregulated the expression of OPG and downregulated the expression of RANKL in KOA subchondral bone measured by real-time PCR and Western blot. (a, b) Real-time PCR analysis of OPG and RANKL. (c–f) Western blot assay of OPG and RANKL. (g) Ratio of OPG/RANKL measured by Western blotting. Values are means ± SEMs. *n* = 6 per group. Compared with the corresponding control group: ^*∗*^*P* < 0.05 and ^*∗∗*^*P* < 0.01; compared with the corresponding model group: ^#^*P* < 0.05 and ^##^*P* < 0.01; compared with the corresponding electroacupuncture group: ^▲^*P* < 0.05 and ^▲▲^*P* < 0.01.

**Figure 6:**
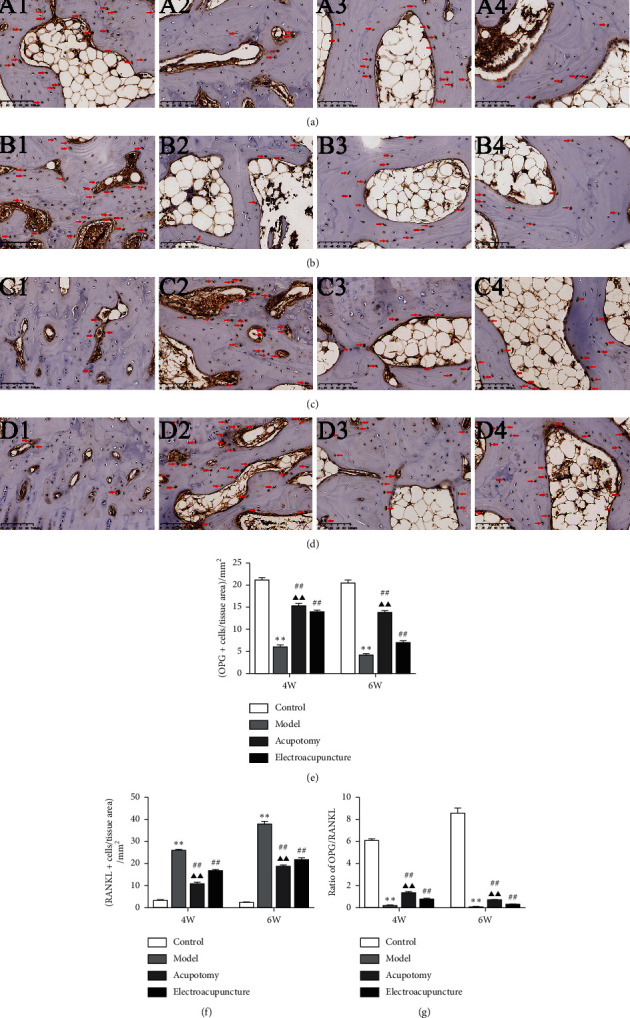
Acupotomy intervention upregulated the expression of OPG and downregulated the expression of RANKL in KOA subchondral bone measured by immunohistochemistry. (a) (b) Immunohistochemical staining of OPG (magnification × 200). A1: 4w control group, A2: 4w model group, A3: 4w acupotomy group, A4: 4w electroacupuncture group; B1: 6w control group, B2: 6w model group, B3: 6w acupotomy group, B4: 6w electroacupuncture group. (c, d) Immunohistochemical staining of RANKL (magnification × 200). C1: 4w control group, C2: 4w model group, C3: 4w acupotomy group, C4: 4w electroacupuncture group; D1: 6w control group, D2: 6w model group, D3: 6w acupotomy group, D4: 6w electroacupuncture group. (e) Expression of OPG-positive cells in subchondral bone. (f) Expression of RANKL-positive cells in subchondral bone. (g) Ratio of OPG/RANKL measured by immunohistochemistry. Values are means ± SEMs. *n* = 6 per group. Compared with the corresponding control group: ^*∗*^*P* < 0.05 and ^*∗∗*^*P* < 0.01; compared with the corresponding model group: ^#^*P* < 0.05 and ^##^*P* < 0.01; compared with the corresponding electroacupuncture group: ^▲^*P* < 0.05 and ^▲▲^*P* < 0.01.

Changes in the expression of OPG and RANKL were quantified using real-time PCR, Western blot and immunohistochemistry. The results of the real-time PCR assay demonstrated that mRNA expression of OPG was significantly decreased (*P* < 0.01, *P* < 0.05) in the 4w/6w model group compared with the corresponding 4w/6w control group, while it was significantly increased (*P* < 0.01) in the 4w/6w acupotomy and electroacupuncture groups compared with the 4w/6w model group. However, acupotomy intervention further effectively promoted the mRNA expression of OPG (*P* < 0.01) compared with electroacupuncture intervention (Figure 5(a)). As expected, mRNA expression of RANKL in the 4w/6w model group was significantly increased (*P* < 0.01) compared with the corresponding 4w/6w control group, while it was significantly decreased (*P* < 0.01) in the 4w/6w acupotomy and electroacupuncture groups compared with the 4w/6w model group. Acupotomy intervention further effectively suppressed the mRNA expression of RANKL (*P* < 0.01, *P* < 0.05) compared with electroacupuncture intervention (Figure 5(b)). The immunohistochemical staining of OPG and RANKL further confirmed the results (Figure 6). The expression levels of OPG and RANKL proteins were consistent with those of mRNA results between the 4w/6w model and control groups. Only OPG protein expression was significantly upregulated (*P* < 0.05) between the 4w acupotomy and 4w model group, while there was no difference (*P* > 0.05) between the 6w acupotomy group and 6w model group. There was no difference (*P* > 0.05) in OPG protein expression between the 4w/6w electroacupuncture group and the 4w/6w model group. OPG protein expression also did not significantly differ (*P* > 0.05) in the 4w/6w acupotomy and electroacupuncture groups (Figures 5(c)–5(e)). RANKL protein expression was significantly decreased (*P* < 0.01) in the 4w/6w acupotomy and 6w electroacupuncture groups compared with the 4w/6w model group. Only RANKL protein expression was significantly decreased (*P* < 0.05) in the 6w acupotomy group compared with the 6w electroacupuncture group (Figures 5(c), 5(d), and 5(f)). The immunohistochemistry and Western blot results also showed that the ratio of OPG/RANKL significantly decreased in the 4w/6w model group compared with the 4w/6w control group (Figures 5(g) and 6(g)), indicating highly active osteoclasts in KOA subchondral bone and strong bone resorption. The ratio of OPG/RANKL was also significantly increased in the 4w/6w acupotomy compared with the 4w/6w model group. These results suggested that acupotomy inhibition of subchondral bone resorption might be exerted by reducing the activity of osteoclasts via the OPG/RANKL pathway in KOA rabbits.

## 4. Discussion

Subchondral bone lesions play an indispensable effect in the KOA process, which is thought to be crucial for accelerating cartilage degeneration. Under physiological conditions, the subchondral bone serves as a mechanical support for articular cartilage, which can buffer and disperse mechanical stress from the joint to prevent cartilage injury by localized stress concentration [27]. Continuously abnormal mechanical load induced by KOA in the knee joint is the most common initiator of subchondral bone lesions [10]. When this abnormal mechanical load exceeds the physiological endurance of the subchondral bone, it will eventually lead to subchondral bone microfracture and initiate bone remodeling [28]. Subchondral bone lesions will weaken its mechanical strength and the ability to buffer mechanical stress accordingly [29], which increases the risk of cartilage injury and accelerates the KOA process. Previous studies have found that the increased subchondral bone loss was positively correlated with the level of cartilage erosion in KOA [30, 31].

Promoting the repair of damaged cartilage remains the core of KOA therapy. Strategies were proposed to alleviate KOA cartilage degeneration by inhibiting subchondral bone remodeling, including estrogens, bisphosphonates, strontium ranelate, and cathepsin K inhibitors. However, the present clinical efficacy of bone metabolism therapy still has certain limitations. The currently available anti-osteoporotic drugs can improve the radiographic structure of subchondral bone by inhibiting bone resorption to a certain extent, but none of them can retard the progress of KOA in clinical practice [12]. The role of mechanical load in subchondral bone remodeling and injury repair has been of gradual concern. Studies have shown that a suitable mechanical load can effectively inhibit KOA-induced subchondral bone osteoporosis and decrease bone mass [32–34], thereby retarding KOA procession. Based on evidence showing that mechanical loads were involved in the pathogenesis in bones and regulated bone remodeling [35, 36], biomechanical therapy has become an emerging method for KOA treatment by affecting subchondral bone. However, whether acupotomy as biomechanical therapy is sufficient to prevent or reverse KOA-induced subchondral bone lesions has not been directly investigated. The biomechanical effects of acupotomy have been confirmed in our preliminary studies [21–24]. It is well known that muscle dysfunction, characterized by weakened muscle strength and reduced coordination of muscle groups, is a crucial cause of KOA knee mechanical imbalance [37]. We chose tendons around the knee as the acupotomy intervention points to correct the abnormal mechanical environment in the knee joint by regulating the mechanical function of muscle-tendons, followed by effectively activating integrin *β*1 [22], the crucial mechanical receptor of chondrocytes, and the downstream FAK-PI3K mechanical signaling pathway [21], which inhibited the degradation of the chondrocyte extracellular matrix and promoted chondrocyte proliferation in KOA rabbits [24]. Can this intra-articular benign mechanical load ameliorate KOA subchondral bone lesions through the OPG/RANKL mechanical signaling pathway after acupotomy intervention? Does the inhibition of benign mechanical loading on subchondral bone remodeling contribute to chondroprotective effects of acupotomy?

Immobilization of the knee joint is a widely used method for KOA model induction [38], which has been applied in research on acupuncture treatment of KOA. In previous studies, we found that immobilization of the knee joints of rabbits induced joint rigidity, as measured by passive range of motion (PROM), and the cartilage eroded integrally with extensive bone marrow edema in subchondral bone as observed by MRI [24], accompanied by reduced expression of COL-II and Aggrecan in cartilage [21]. Further, cartilage oligomeric matrix protein (COMP), the biomarker of KOA cartilage degradation, markedly increased in the serum of rabbits [24], which was consistent with the changes in serous COMP observed in KOA patients [39]. In this study, obvious cartilage erosion, significant deletion of Safranin O staining, and subchondral bone destruction were observed after 4 or 6 weeks of immobilization. These indicators suggested that OA-like cartilage lesions and subchondral bone destruction could be duplicated in rabbits with immobilized knee joints similar to the phenomena observed in human clinical cases (Figure 1).

Evidence shows that cartilage surface defects, deletion of Safranin O staining, and disorder of the chondrocyte arrangement, as determined by morphological observation, gradually worsened in KOA cartilage induced by immobilization for 4 and 6 weeks; these symptoms were accompanied by a continuous increase in the Mankin score, indicating the rapid development of KOA. We found that both the acupotomy and electroacupuncture intervention successfully protected against cartilage degeneration and effectively decreased the Mankin score. However, the enhancement of correction of the abnormal mechanics by acupotomy seemed to be more effective in slowing cartilage erosion, and neither intervention could fully reverse the pathological changes in cartilage. These data further support the direct effect of acupotomy and electroacupuncture on promoting KOA cartilage repair (Figure 1).

As reported previously in numerous studies, enhanced bone resorption was the most typical pathological characteristic of KOA subchondral bone [40], which was manifested by a marked decrease in Tb.N and BV/TV and a marked increase in Tb.Sp [34, 41]. The studies clarified that ameliorating the subchondral bone structure of KOA by inhibiting the decrease in Tb.N and BV/TV and the increase in Tb.Sp was an effective approach to relieve KOA cartilage degeneration [42, 43]. HE staining was used to probe into the effects of acupotomy intervention on bone structure [43]. The obvious subchondral bone destruction in the Model group showed that Tb.Ar, Tb.Pm, Tb.N, and BV/TV were significantly decreased and Tb.Sp was significantly increased. Moreover, by observing the subchondral bone changes in the KOA model induced by 4 or 6 weeks of immobilization, bone destruction gradually increased. Further research of bone structural properties proved that acupotomy or electroacupuncture-treated rabbits had observably increased BV/TV and Tb.N and decreased Tb.Sp compared with the rabbits in the Model group. However, whether in the 4- or the 6-week group, the effect of acupotomy on subchondral bone destruction inhibition was superior. To our knowledge, this is the first study to show that acupotomy intervention in immobilization-induced KOA rabbits improves the morphometric parameters of subchondral bone, suggesting that acupotomy can inhibit KOA subchondral bone loss. According to the pathological Mankin score of cartilage in each group, the reduction of subchondral bone loss was accompanied by the improvement of cartilage damage, suggesting that acupotomy intervention alleviated KOA cartilage degeneration by inhibiting subchondral bone lesion. Compared with electroacupuncture, acupotomy shows a superior effect in suppressing KOA subchondral bone remodeling by correcting the abnormal mechanical load in the joint (Figure 2).

What are the underlying mechanisms of acupotomy intervention in suppressing subchondral bone lesions? The initiation of KOA subchondral bone remodeling is coupled with disruption of the balance between bone resorption and bone formation [44]. Osteoclasts are first activated to enhance bone resorption in the damaged bone area [3, 45]. Subchondral bone loss, characterized by decreased Tb.N and BV/TV and increased Tb.Sp, is the secondary result of hyperactive bone resorption by osteoclasts in early-stage KOA [46]. Activating osteoclasts is a central event and driving force in the pathogenesis of subchondral bone lesion, while inhibiting osteoclast activity contributes to preventing bone resorption and KOA procession [40, 47]. By combining with RANK, RANKL promotes osteoclast maturation and differentiation, and it activates signaling pathways related to osteoclastogenesis, thus promoting the expression of osteoclast-specific genes including TRAP, MMP-9, and Ctsk [48]. Studies have shown that, by downregulating the expression of MMP9, TRAP [49], and Ctsk [50], osteoclastogenesis can be inhibited and osteoclast activity can be confined to reduce KOA subchondral bone resorption. In our study, osteoclasts were typically activated, showing that the expression of TRAP and MMP-9-positive cells and Ctsk mRNA were significantly increased in subchondral bone of KOA rabbits. In addition, osteoclast activity continued to increase with extension of the immobilization, which was consistent with the variation in morphometric parameters of subchondral bone. Further analysis of the osteoclast activity indicated that acupotomy or electroacupuncture-treated rabbits had a significant reduction of TRAP and MMP-9-positive cells and Ctsk mRNA expression. In both the 4-week and 6-week immobilization-induced KOA rabbits, acupotomy was more advantageous in confining osteoclast activity than electroacupuncture. Our results further corroborated the finding that acupotomy intervention was sufficient to inhibit KOA subchondral bone lesions by downregulating the expression of MMP9, TRAP, and Ctsk to suppress osteoclast activity (Figure 4).

To further confirm that acupotomy suppressed bone resorption, expression of the osteoblast-specific gene OCN was examined. Osteoblasts are essential for bone formation, and OCN is indispensable for osteoblast differentiation [51]. The content of OCN is a direct response to osteoblast activity and the bone formation rate. Although osteoblasts were compensatorily activated in KOA subchondral bone, it was completely insufficient to reverse the continuous increase in hyperactive osteoclasts. Furthermore, with an extension of immobilization, osteoblast activity continued to decrease, as reflected by the reduction of OCN expression, suggesting that the balance between bone resorption of osteoclasts and bone formation of osteoblasts was broken in immobilization-induced KOA rabbits. A previous study has indicated that low expression of OCN is observed in KOA subchondral bone with severe bone loss, resulting in a reduction of bone formation [52], while increasing the content of OCN can effectively promote osteoblast activity in damaged bone [53]. Our study demonstrated that acupotomy and electroacupuncture effectively promoted bone formation by activating OCN expression. In both 4-week and 6-week immobilization-induced KOA rabbits, acupotomy was more advantageous in promoting osteoblast activity than electroacupuncture. Our results further corroborate the finding that acupotomy intervention was superior for restoring the dynamic balance of osteoclastic bone absorption and osteoblastic bone formation in KOA subchondral bone (Figure 3).

How acupotomy intervention regulates osteoclast activity remains to be determined. The maturation, differentiation, and osteoclast activity were mediated by the OPG/RANKL pathway. The actions of OPG and RANKL in damaged subchondral bone have been closely investigated since elevated expression of RANKL and declining expression of OPG have been observed in KOA [54]. Studies have revealed that blockade of the increase in RANKL and decrease in OPG protects against hyperactive osteoclasts and inhibits bone resorption [55, 56]. In addition, the OPG/RANKL ratio is one of the best characterized biomarkers associated with the bone destruction pathology [57]. As reported previously in numerous studies, OPG/RANKL is a mechanically sensitive signaling pathway [58, 59]. Benign mechanical stimulation can increase the OPG/RANKL ratio in KOA subchondral bone, which effectively inhibits osteoclast activity and bone resorption [60]. Our previous studies [21] have confirmed that acupotomy intervention activates the FAK-PI3K mechanical pathway to relieve KOA cartilage degeneration by correcting abnormal mechanical stress. However, whether this benign mechanical stimulation can promote subchondral bone recovery from KOA-induced bone resorption by the OPG/RANKL pathway remains to be explored. In our study, increased expression of RANKL mRNA and protein and decreased expression of OPG mRNA and protein were measured in KOA subchondral bone. Immobilization for 6 weeks revealed higher RANKL and lower OPG expression, suggesting an enhancement of osteoclast activity and stronger bone resorption. The results obtained with real-time PCR and immunohistochemical staining showed that acupotomy or electroacupuncture-treated rabbits had significantly decreased expression of RANKL and markedly increased expression of OPG. However, the Western blot results showed that acupotomy intervention upregulated the decrease in OPG protein and downregulated the increase in RANKL protein, while electroacupuncture intervention had a poor effect. As expected, the OPG/RANKL ratio determined by Western blotting and immunohistochemistry was roughly consistent with the expression of the osteoclast-specific genes TRAP, MMP-9, and Ctsk. These data confirmed that the benign mechanical stress after acupotomy intervention effectively inhibited the increase in RANKL and the decrease in OPG of KOA subchondral bone. Acupotomy, a biomechanical therapy, can extensively confine hyperactive osteoclasts via the OPG/RANKL mechanical signaling pathway to inhibit bone resorption in KOA subchondral bone (Figures 5 and 6).

The present study has the following limitations. First, a positive drug group was not set as the control group for acupotomy, but rather the electroacupuncture group. Based on many reports [12, 20], bone metabolism regulators represented by bisphosphonates can only inhibit subchondral bone resorption in the KOA animal model, and the efficacy in the clinic has been disappointing. Considering the use of KOA subchondral bone as the entry point to explore the mechanism of acupotomy treatment of KOA from the biomechanical perspective and to comprehensively evaluate the operability in this study, electroacupuncture may be the most appropriate control group at present. Electroacupuncture, as a usual and effective therapy, is also a nonsurgical and nonpharmacological intervention to treat KOA. Moreover, the position of the intervention points of the electroacupuncture and acupotomy is close. If there is a more feasible biomechanical therapy that can be used as a control group in the future, we will continue to conduct in-depth research. Second, we successfully established the KOA model by 6-week immobilization in the previous study, which was consistent with early and mid-stage KOA lesions. In the current study, in view of the dynamic pathological changes of subchondral bone in the development of KOA, we established immobilization-induced KOA models for 4 and 6 weeks to evaluate the therapeutic effects of acupotomy on subchondral bone lesions from the onset of KOA to early and mid-stage KOA. In subsequent studies, we should continue to extend the duration of knee immobilization to 8 or even 12 weeks to dynamically observe the regulatory effect of acupotomy on subchondral bone remodeling during the entire pathogenesis of KOA.

## 5. Conclusion

In summary, we found that acupotomy inhibited osteoclast activity and promoted osteoblast activity to ameliorate hyperactive subchondral bone resorption and relieve cartilage degeneration in immobilization-induced KOA rabbits, which may be mediated by the OPG/RANKL signaling pathway. The mechanical effect of acupotomy exerts chondroprotective effects by mitigating KOA subchondral bone lesions, which might supply original insights into the treatment of KOA with acupotomy and a novel therapeutic approach in the biomechanical treatment of KOA.

## Data Availability

The data supporting the conclusions of this study are available from the corresponding author upon reasonable request.
